# Use of speleotherapy in patients with post-COVID-19 syndrome

**DOI:** 10.3389/fmed.2025.1566235

**Published:** 2025-06-13

**Authors:** René Garbsch, Mona Kotewitsch, Hendrik Schäfer, Marc Teschler, Frank C. Mooren, Boris Schmitz

**Affiliations:** ^1^Department of Rehabilitation Sciences, Faculty of Health, University of Witten/Herdecke, Witten, Germany; ^2^DRV Clinic Königsfeld, Center for Medical Rehabilitation, Ennepetal, Germany

**Keywords:** SARS-CoV-2, post-COVID-19, long COVID, speleotherapy, DLCO

## Abstract

**Background:**

The post-COVID-19 syndrome (PCS) is characterized by persistent or newly developed symptoms and performance deficits lasting at least 3 months following SARS-CoV-2 infection, with dyspnea as a common symptom. Speleotherapy, a form of climatotherapy utilizing the microclimatic conditions of natural or artificial caves, has been proposed as a supportive treatment for chronic airway diseases, potentially improving lung function and exercise tolerance.

**Methods:**

This study investigated the short-term effects of speleotherapy on lung diffusion capacity (DLCO) in PCS patients through a prospective interrupted time-series analysis. Forty-six patients (51.9 ± 9.3 years; 43% female) referred for rehabilitation were included, with a history of COVID-19 infection and persistent deficits lasting over 3 months. Patients underwent spirometric assessments of DLCO repeatedly on days without speleotherapy intervention and on days with intervention, alongside subjective symptom evaluations using the Nijmegen questionnaire.

**Results:**

PCS patients performed a median of four out of seven speleotherapy sessions during rehabilitation, resulting in a total of 388 measurements. Analysis revealed no significant changes in DLCO or related parameters (transfer coefficient (KCO), inspiratory volume (IV), total lung capacity (TLC), Residual volume (RV)) during rehabilitation and between speleotherapy and control days (*p* ≥ 0.544). Subgroup analysis of patients with DLCO below 80% of predicted reference and symptom severity assessments also revealed no therapeutic benefits. Speleotherapy frequency showed no dose-dependent effects on pulmonary outcomes (*p* = 0.171). Findings from a small control group confirmed these results (*p* ≥ 0.997).

**Conclusion:**

Speleotherapy did not improve DLCO or alleviate symptoms in PCS patients within this study cohort. Further research is needed to investigate whether speleotherapy can alleviate pulmonary dysfunction in different PCS populations.

The post-COVID-19 syndrome (PCS) occurs after a SARS-CoV-2 infection and is characterized by persistent or newly expressed symptoms and performance deficits lasting for at least 3 months, including dyspnea as one lead symptoms (1, 2). Spa-based interventions, including balneotherapy or mineral water inhalations, have been used to improve lung function and to alleviate respiratory symptoms in chronic airway diseases (3) and have been discussed as potentially beneficial in PCS rehabilitation (4). To this end, recent studies have reported initial positive effects, including reductions in fatigue, musculoskeletal pain, and respiratory symptoms and improvements in overall functional capacity in PCS patients (5, 6). Spa-based interventions are hypothesized to improve pulmonary function by enhancing mucociliary clearance, reducing airway inflammation and oxidative stress, and modulating immune responses, that is, mechanisms that may also be relevant for alleviating diffusion capacity impairments commonly observed in PCS patients (7, 8). Speleotherapy, a form of climatotherapy, uses microclimatic conditions of natural or artificial caves for the supportive treatment of chronic airway diseases such as asthma (9, 10) and chronic obstructive pulmonary disease (11, 12). Speleotherapy has been suggested to reduce the disease burden and increase pulmonary function and exercise tolerance in these patients (9–12). Comparable to spa-based interventions, physical and chemical effects of the respective microclimate (low temperature, high relative humidity, and high concentration of minerals) could lead to a (short-term) improvement in lung diffusion capacity (DLCO) presumably by reducing the surface tension at the air–water interface in the alveoli with effects on oxygenation (13). However, data on spa-based interventions targeting pulmonary sequelae remain scarce, whereas reports on speleotherapy for treating diffusion impairments in PCS are missing from the literature.

In this study, we investigated the short-term effects of speleotherapy on DLCO as the primary outcome variable using a prospective interrupted time-series analysis (14), with diffusion capacity assessed repeatedly in paired blocks comprising one measurement directly after speleotherapy and another on the following day without speleotherapy. Inclusion criteria were a history of (at least one) COVID-19 infection and ongoing or newly expressed performance deficits lasting for at least 3 months prior to recruitment. All PCS patients were eligible to participate regardless of pulmonary function, with the requirement to participate in at least two speleotherapy sessions during standard rehabilitation described in detail elsewhere (15). Patients who were unable to undergo speleotherapy due to anxiety, etc., served as a control group. Spirometric assessment, including measurements of forced vital capacity and single-breath lung diffusion (COSMED Quark PFT, Rome, Italy, CV = 13.84%), was performed at admission 2–3 days before the first therapeutic session in PCS patients referred to inpatient medical rehabilitation at Clinic Königsfeld, Germany, between February 2023 and November 2024. Potential short-term effects were assessed by measuring pulmonary diffusion capacity for carbon monoxide immediately after speleotherapy (within 1 h) and comparison with assessments on the following day without speleotherapy (>18 h after therapy). Speleotherapy sessions were conducted in the “Heilstollen Ennepetal” (Kluterthöhle, 51 °17′57.2″N 7°21′15.5″E, a reef limestone cave), included in the “Deutscher Bäderkalender” in 1954 with the following microclimate conditions. Air composition: 77.5 vol.% nitrogen, 19.9 vol.% oxygen, 0.13 vol.% carbon dioxide, and <0.01 vol.% hydrogen with temperatures ranging from 9.9 to 11.1°C and relative humidity levels of 96–99% (Institut Prof. Dr. Jäger GmbH, Tübingen, Deutschland). Patients were seated in reclining chairs at a depth of 40 m and performed speleotherapy for 120 min per session, with sessions scheduled at least twice a week. During the sessions, patients were instructed to relax and focus on breathing. The Nijmegen questionnaire (16, 17) was asked at each spirometric assessment to investigate subjective effects on disease burden, in particular on respiratory symptoms. Power analysis suggested a sample size of *N* = 42 to detect a mean difference in diffusion capacity on days with and without speleotherapy with a power of 1 − β = 0.8 at α = 0.05, given an effect size *d* = 0.8 (18). Statistical analyses were performed using SPSS (V.28, IBM, Armonk, United States) and GraphPad Prism (V.10, GraphPad Software, Boston, United States). The study was approved by the local ethical review committee (Ethik-Kommission Universität Witten/Herdecke; reference number 159/2021). Patients provided data from their electronic health record at the end of rehabilitation as defined in artice 20 of the the European General Data Protection Regulation (GDPR).

PCS patients (N = 46; 51.9 ± 9.3 years; 43% female) were referred to rehabilitation 478.7 ± 231.8 days after (first) acute infection. Fatigue/exercise intolerance was the most prevalent leading symptom observed in 98% of patients, followed by shortness of breath (83%) and cognitive dysfunction (78%). During the acute phase of infection, 94% of patients received ambulant care or acute care at home, whereas 6% of patients required in-hospital care. In addition, patients reported a high frequency of endocrine, nutritional, and metabolic disorders and circulatory system disorders (data not shown), comparable to the general PCS patient characteristics at our clinic (15, 19, 20). At admission, PCS patients presented with pulmonary function within reference including forced vital (inspiratory) capacity, forced expiratory volume, forced and peak expiratory flow, and maximum expiratory and inspiratory flow (all ≥ 90% of predicted reference), whereas mean diffusing capacity for carbon monoxide (DLCO) was at 20.5 ± 4.7 mL/min/mmHg equal to 77.1 ± 13.5% of predicted reference. The transfer coefficient (KCO) for DLCO and alveolar volume (VA) was 4.0 ± 0.6 mL/min/mmHg/L corresponding to 92.7 ± 15.7% of predicted reference, whereas inspiratory volume (IV) at 3.6 ± 0.9 L, total lung capacity (TLC) at 5.4 ± 1.2 L, and residual volume (RV) at 1.3 ± 0.8 L were limited at ≤ 80% of predicted reference.

Overall, patients performed a median of 4 (7) speleotherapy sessions during 3–4 weeks, resulting in a total of 194 therapy and 194 corresponding control assessments (n = 388 measurements). No significant changes in DLCO and KCO were observed (pre–post-test, *p* ≥ 0.886), and assessments directly after speleotherapy were comparable to assessments on control days for DLCO (therapy 20.2 ± 5.5; control 20.6 ± 5.5; *p* = 0.544) and KCO (therapy, 4.0 ± 0.7; control, 4.0 ± 0.6; *p* = 0.932) (Figures 1A,B). Additional analysis exclusively in patients with a significant limitation in DLCO (*N* = 22; *n* = 184 measurements), defined as less than 80% of predicted reference (21) in baseline DLCO assessment, also did not reveal any effect (all *p* ≥ 0.641) (Figures 1C,D). Questionnaire results of symptom severity, including faster or deeper breathing, shortness of breath, tightness in the chest, and not being able to take a deep breath, also indicated no difference between therapy and control days (*p* = 0.635). Of note, Spearman’s rank correlation analysis did not reveal a dose effect in terms of the frequency of speleotherapy and change in DLCO (pre–post-test, *p* = 0.171). Comparison to the control group (N = 6 patients; 27 assessments) revealed no significant between-group interaction effect for changes in DLCO or KCO over time (both *p* ≥ 0.997).

**Figure 1:**
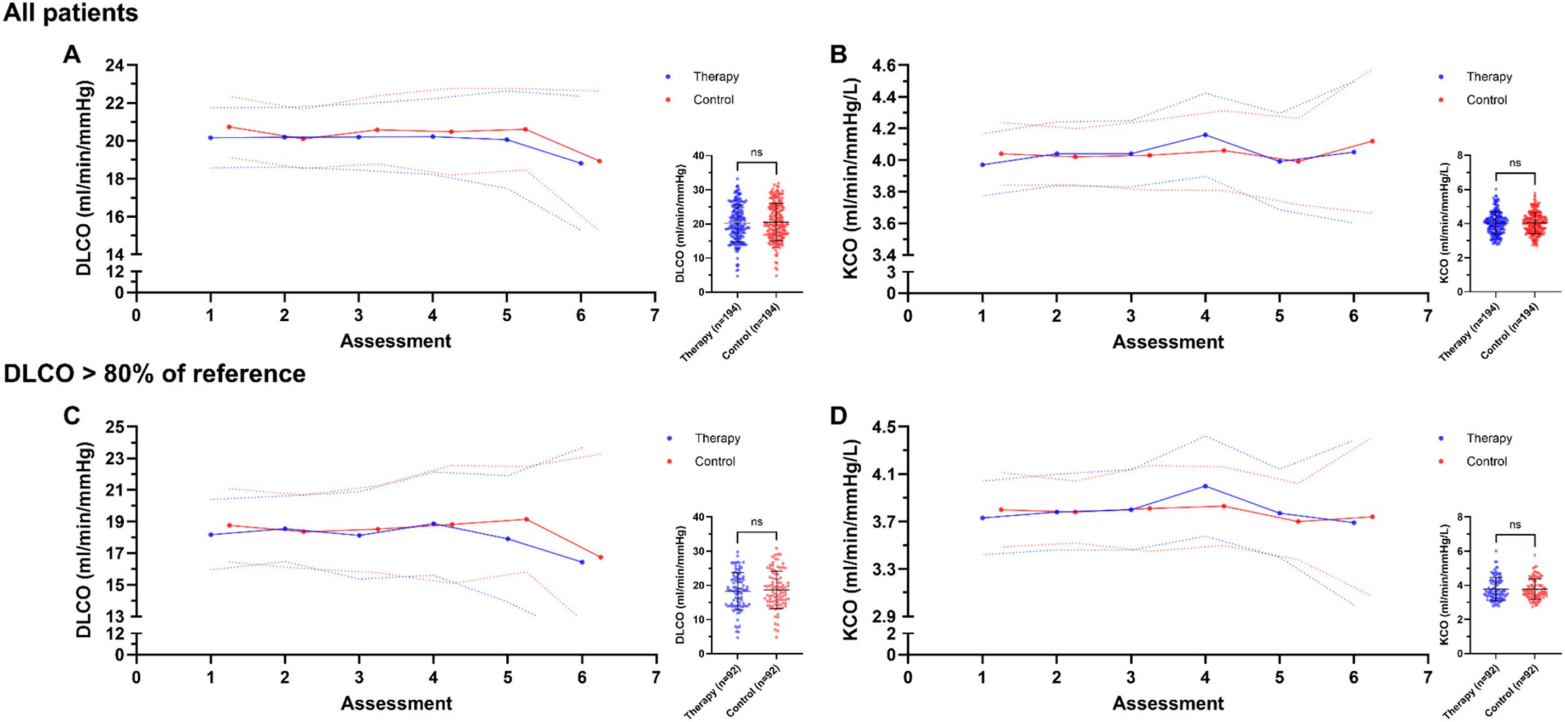
Speleotherapy does not affect lung diffusing capacity and gas transfer ability in patients with post-COVID-19 syndrome (PCS). **(A,B)** Diffusing capacity for carbon monoxide (DLCO) and transfer coefficient (KCO) over the rehabilitation process on days with speleotherapy (*blue*) and days without speleotherapy (*red*) for all PCS patients (*N* = 46). **(C,D)** DLCO and KCO values over the rehabilitation process for patients (*N* = 22) with restricted lung diffusing capacity (<80% of predicted reference). Assessments were performed after speleotherapy and on control days (>18 h after therapy). Data are presented as mean with 95% confidence interval and were compared by two-way repeated measures ANOVA. Trend line was modeled using linear regression slopes. Inserts show scatter plots (mean ± SD) of individual measurements of all therapy and control assessments compared by unpaired *t*-test.

To the best of our knowledge, this study is the first to report on the effects of speleotherapy in PCS patients. Our observations in a representative medium-sized cohort did not reveal any significant effects of speleotherapy on diffusion capacity in PCS patients. In addition, no effects on KCO, IV, TLC, RV, and perceived symptoms were detected. In this trial, DLCO was selected as the primary outcome variable due to the fact that a substantial proportion of patients with PCS present with impaired lung diffusing capacity, reflecting long-term deficiency in alveolar–capillary gas exchange after an acute COVID-19 infection. A recent review showed a prevalence of 30.5–39.8% for abnormal diffusion capacity (22), an impairment that has been associated with dyspnea, reduced exercise capacity, and diminished quality of life (23, 24), making DLCO a meaningful diagnostic variable for evaluating therapeutic interventions. In addition, it has been suggested that already low-grade reductions in DLCO below 80% of reference, even in the presence of otherwise normal pulmonary functions, may be significantly associated with fatigue in PCS and other diseases (25–27). Although speleotherapy has shown potential benefits in patients with chronic respiratory diseases, its physiological effects in PCS are unknown. Our findings do not indicate measurable improvements in diffusion capacity, suggesting that the specific microclimatic conditions in the reef limestone cave may not influence relevant mechanisms in PCS. However, subjective reports from patients during weekly visits were consistently positive, even though this was not reflected in the questionnaire results. One possible explanation may be the extended relaxation periods during speleotherapy sessions, which could have contributed to improved fatigue management. Given the lack of prior studies in this population and several limitations of the present study, the results should be interpreted with caution. Although overall dose–response analyses showed no significant correlation between changes in diffusion capacity and number of therapy sessions, the total number of speleotherapy sessions varied considerably across participants from two to nine sessions. It should also be mentioned that the time after acute infection varied considerably between patients, even though this factor has not been identified as a relevant effector of pulmonary outcomes in previous analyses (15, 19). Of note, most participants had preserved lung function at baseline, which may have limited the potential for detecting improvements. However, a subgroup analysis of patients with impaired DLCO also showed no measurable effects. Finally, the study may be limited by the absence of a larger control group, which may affect the interpretability and strength of conclusions regarding the effects of speleotherapy. It also needs to be noted that PCS patients in this study were able to participate in medical rehabilitation in general, and findings may not be generalizable to patients with more severe symptoms or different organ manifestations. In particular, subgroups with more pronounced pulmonary involvement, such as those with persistent radiological abnormalities, marked DLCO reduction, or ongoing need for oxygen therapy, may respond differently, which should be addressed in future studies. Our findings should also be viewed in the broader context of respiratory rehabilitation strategies for PCS. Non-pharmacological approaches, including spa-based interventions such as mineral water inhalations, have been discussed as supportive options, particularly in post-acute recovery phases (4). Although our study did not demonstrate an effect on diffusion capacity, recent studies suggest that such interventions may improve fatigue, musculoskeletal pain, respiratory symptoms, and overall functional capacity in PCS patients (5, 6), highlighting their potential role within multimodal rehabilitation concepts.

## Data Availability

The raw data supporting the conclusions of this article will be made available by the authors, without undue reservation.
